# Case of a Still-Born Child Restored to Life

**Published:** 1807-10

**Authors:** J. C. Rousseau

**Affiliations:** Philadelphia


### *Case of a still-born Child restored to Life.* By J. C. Rousseau, M. D. of Philadelphia.

On the 21st of March last, I was desired to attend Mrs. O****, come to the full time of her pregnancy. Her labour began with a violent flooding, which being, by proper means, stopped, every thing progressed regularly.

The head of the child having at last engaged in the pelvis, and beginning to make its appearance at the os externum, I gave her hopes of a prompt delivery; upon which she informed me, that she had never been, in any previous ous labour, delivered in bed; and it was her wish to, and she immediately did, place herself kneeling down at the foot of the bed.

Her labour being quick, the child was soon born, and as I suspected from the flooding, without any sign of life. I had hardly any time to turn the child in my hands, when, without any warning, she jumped into her bed, leaving a lifeless child in my hands, with its placenta pulled out upon the floor.

My feelings upon this embarrassing occasion may be more easily felt than described. The mother had the first right to my cares; the child's life was in the utmost danger: there was no alternative. I went to the mother, after having delivered up the child to an assistant; and, at the same time called for warm water, which fortunately was at hand. Finding no flooding, and every thing in a fair way with the mother, I returned immediately to the child.

It gave no sign of life; but finding the pulsation strong in the cord, and persuaded, as I have always been, that an organic life exists a long time after birth, between the child and placenta, even when detached from the uterus, I immersed both child and placenta in a tub of warm water, and then stooping over the child, and placing one of my hands on his back, and the other on its breast, I enclosed his nose and mouth in mine, began to inflate its lungs, and expel the air alternately by the pressure of my hands.

I continued this operation for three quarters of an hour, before the child exhibited any signs of life, stopping now and then to observe whether I could perceive any natural motion of the thorax. It was not long after, when the thorax began to move, and the child inspired once. About half a minute elapsed between the first and second inspiration. This interrupted life, if I may so call it, lasted better than a quarter of an hour, during which time I stimulated the nose and fauces of the child. The respiration becoming quicker and quicker, I began to stop gradually by pressure of my finger on the cord, the pulsation that was still very strong, and in a short time I separated the child from the placenta, and had the satisfaction to see both mother and child perfectly free from danger.

This child is now as hearty and robust as any other child of its age, appearing to have received no injury from such an uncommon entrance into life.

*July* 29, 1805.

